# Energy/Area-Efficient Scalar Multiplication with Binary Edwards Curves for the IoT

**DOI:** 10.3390/s19030720

**Published:** 2019-02-10

**Authors:** Carlos Andres Lara-Nino, Arturo Diaz-Perez, Miguel Morales-Sandoval

**Affiliations:** 1 CINVESTAV Tamaulipas, Victoria 87130, Mexico; mmorales@tamps.cinvestav.mx; 2 CINVESTAV Guadalajara, Zapopan 45019, Mexico; adiaz@cinvestav.mx

**Keywords:** elliptic curve cryptography, low-power, low-energy, binary Edwards curves, scalar multiplication, internet of things, wireless sensor networks, lightweight cryptography

## Abstract

Making Elliptic Curve Cryptography (ECC) available for the Internet of Things (IoT) and related technologies is a recent topic of interest. Modern IoT applications transfer sensitive information which needs to be protected. This is a difficult task due to the processing power and memory availability constraints of the physical devices. ECC mainly relies on scalar multiplication (*kP*)—which is an operation-intensive procedure. The broad majority of *kP* proposals in the literature focus on performance improvements and often overlook the energy footprint of the solution. Some IoT technologies—Wireless Sensor Networks (WSN) in particular—are critically sensitive in that regard. In this paper we explore energy-oriented improvements applied to a low-area scalar multiplication architecture for Binary Edwards Curves (BEC)—selected given their efficiency. The design and implementation costs for each of these energy-oriented techniques—in hardware—are reported. We propose an evaluation method for measuring the effectiveness of these optimizations. Under this novel approach, the energy-reducing techniques explored in this work contribute to achieving the scalar multiplication architecture with the most efficient area/energy trade-offs in the literature, to the best of our knowledge.

## 1. Introduction

The deployment of Internet of Things (IoT) applications is pushing society to interact with smart environments on a regular basis. Smartphones, buildings, vehicles, roads, home appliances; most new instances of these technologies are being equipped with capabilities for data sensing and internet connectivity [1]. The data retrieved by these systems might be sensitive, since it can be inherently confidential [2] or can be used to infer a user’s behavior [3]. Providing security for the IoT is said to be the equivalent of providing security for a conventional network, with the added complexity that the network can be physically reached by attackers [4].

A common characteristic in many IoT nodes is that they suffer from physical constraints, most notably on size and energy [4,5]. For reducing manufacturing costs, devices’ physical size needs to be decreased.

For some, one of the most precious resources of a constrained device is energy [6,7]. The reasoning is that after deployment, some nodes rely on battery systems which cannot be replaced and ought to last for several months or years. That is why “to minimize energy consumption, lightweight Public-key Cryptography (PKC) implementations are a fundamental requirement” [8]. For both cases, lightweight cryptography can provide an effective solution that (a) is physically small and (b) has low energy consumption.

Elliptic Curve Cryptography (ECC) has proven to be one of the best PKC alternatives for constrained applications [8,9,10,11,12,13] where multiple restrictions are also observed. Compared to other PKC systems, ECC features reduced key sizes for equivalent security levels [14]. ECC can be used for achieving key establishment, encryption, authentication, and signatures, among other security functions. The fundamental operation required in ECC is *kP*—which relies on a huge amount of field operations. Although improving the performance and area of this algorithm has been widely addressed in the literature, the energy profile of these systems has been seldom studied [15].

The most popular strategy for reducing the energy consumption of an implementation is to reduce its runtime [16,17,18,19,20]. However, some performance-enhancing techniques might prove to be too costly for constrained devices in terms of hardware utilization. Area minimization can also lead to energy savings by reducing the power dissipation—to a lesser extent this approach has also been studied in the literature [21,22]. Experimenting with the tradeoffs of both methodologies can lead to novel design insights which can be used to reduce the energy footprint of the system.

In this paper, we explore the area/energy tradeoffs on a FPGA-based realization of the multiplication scalar for Binary Edwards Curves (BECs) [23]. We use a lightweight area-oriented *kP* architecture as the starting point for applying a sequence of energy-related improvements. The efficiency of these modifications is assessed at each step. Our approach emulates an incremental development, where in the final step a solution which is efficient in both hardware usage and energy consumption is obtained. We have chosen BECs as case study, but the energy-reduction strategies applied can be translated to any other elliptic curves. Our optimizations focus on hardware since in that way the module can be used as a security accelerator which is enabled upon request to further save energy. The proposed improvements are evaluated with performance and area metrics, as well as with a novel method for estimating the energy savings in relation to area costs. Under this novel approach, we show that one of the *kP* architectures proposed—to the best of our knowledge—is the most efficient design reported. As evaluation platform we use the xc6slx16 low-cost FPGA operating at commonly used frequencies.

Our contributions can be summarized as follows:

1. The detailed implementation and assessment of energy-reducing techniques are presented. The techniques employed are often used in the literature, but the actual effectiveness of each one is seldom explored. In this paper we aim at filling this gap by providing detailed implementation results. With this study, researchers aiming at producing new low-energy designs can have a precedent for choosing the strategies best suited to their projects.
2. Our architectures improve the state of the art in regards to area/energy efficiency. This is in part thanks to the carefully designed cryptosystem, and to the followed design methodology.
3. We have created and described a novel evaluation metric for assessing the efficiency of the proposed architectures in terms of energy reduction and area increments. This metric can account for variations in the measurement units, the operational frequency, and the underlying finite field. Thanks to these points we were able to employ the novel metric for benchmarking our architectures and the entirety of the state of the art for low-power/low-energy scalar multiplication realizations.

The rest of the paper is structured as follows. Section 2 briefly enumerates some preliminary notions regarding the topics in this paper. Section 3 describes the energy-oriented improvements applied to ECC architectures in selected works from the literature. The description and implementation for our energy improvements can be found in Section 4. A novel evaluation method for energy improvements is detailed in Section 5. Lastly, our concluding remarks are available in Section 6.

## 2. Preliminaries

### 2.1. Elliptic Curve Cryptography

An elliptic curve can be described as the set of points that satisfy the Weierstrass model in (1) over the finite field $F_{q}$.

(1) $$E:y^{2}+a_{1}xy+a_{3}y=x^{3}+a_{2}x^{2}+a_{4}x+a_{6}\;\mathrm{with}\;a_{i}\in F_{q}$$

Simplifications of (1) and equivalences are used as the basis for different elliptic curve families: random prime, random binary, Koblitz, Montgomery, Edwards, twisted Edwards, binary Edwards, among others.

The elliptic curve points *E*, a group operation + and the point at infinity O form an elliptic curve group *E*($F_{q}$), which can be used in cryptographic applications. The operation + is the addition of points, it varies for each elliptic curve family. Thus *kP* represents the consecutive application of the group operation *k* times over the base point or generator *P*:

(2) Q=P+P+…+P=kP.

In practice *kP* relies on point addition (P+P) and doubling (2P), where each is composed of multiple field operations. The complexity of *kP* depends on the group and field arithmetic definitions. The *kP* calculation is used in any ECC-based algorithm, hence improving its efficiency is critical.

The BECs family is defined by the model

(3) $$E_{B}:d_{1}\left(x+y\right)+d_{2}\left(x^{2}+y^{2}\right)=xy+xy\left(x+y\right)+x^{2}y^{2}$$

where $d_{1},d_{2}\in F_{2^{m}}$ with $d_{1}\neq 0$ and $d_{2}\neq d_{1}^{2}+d_{1}$. These curves are birationally equivalent to binary generic curves [23]. Their principal advantages of BECs are that (a) their group operation is complete, so no extra checks are required and (b) their group operation requires less field operations.

In [23] the authors introduced the concept of *w* coordinates for BEC. By using this point representation it is possible to reduce the amount of field operations required in performing *kP*. Furthermore, the use of projective-*w* coordinates enables reducing the number of inversions required—inversions are some of the most expensive field operations. Differential addition and doubling formulae can be combined with projective-*w* coordinates to achieve the smallest requirements in terms of field operations for *kP* in BECs [24].

### 2.2. Power and Energy

Let the energy (*ENE*) consumed by a circuit to perform a task as the product between the dissipated power (*POW*) and the runtime (*t*):

(4) ENE=POW×t

This approach is employed in multiple works from the literature [17,18,25,26,27].

We consider the runtime to be directly linked with the performance of the system: lower runtime equals higher performance and *vice versa*. The runtime is the product of the latency clock cycles (*LAT*) and the inverse of the operational frequency (*f*):

(5) $$t=LAT\times \frac{1}{f}$$

*POW* is obtained as the sum of the dynamic (*DP*) and static or quiescent (*SP*) powers:

(6) POW=DP+SP

*DP* is the sum of powers associated with clocking, signals, logic, IOs, and dedicated blocks; this includes the data-dependent power. *SP* is dissipated by the whole FPGA fabric and remains somewhat constant regardless of the implemented circuit. For FPGAs the static power tends to be higher than the dynamic part. Each component is usually modeled as

(7) $$DP=e\times f\times A\;\mathrm{and}\;SP=I_{s}\times V_{cc}$$

where *e* is the average energy spent during one clock cycle per area unit, *A* represents the area of the circuit, $I_{s}$ is the static current consumed from the power supply, and $V_{cc}$ is the supply voltage. The designer has control over the operational frequency, the latency, and the area to influence the energy consumption of the system.

The effects of *f* over *ENE* are not straightforward. If *f* is reduced, then *t* grows and *ENE* rises—as shown in (4)—from the *SP* component in *POW*; if *f* is increased, then *POW* may grow due its *DP* element—see (7)—and the increment of *ENE* follows from (4). Finding the optimal operational frequency for the proposed *kP* architecture is outside of the scope of this work, we do however use two operational frequencies (low vs. high) to study this variation.

So, if we seek to reduce *ENE* we need to find a minimum in the balance between the area and the latency. The former has been the main optimization goal for lightweight cryptography, whereas the latter has generated interest in recent years [28].

Other popular optimizations such as clock gating and datapath insulation aim at mitigating the switching activity of parts of the circuit which are not actively used—these aim at reducing the dynamic power consumption of the circuit.

### 2.3. Percentile Differences

The percentile increment (Δ%) is provided whenever new implementation results are presented. These increments are calculated as the difference between the new ($O_{C_{i}}$) and the previous observation ($O_{C_{i-1}}$), with reference to $O_{C_{i-1}}$:

(8) $$\Delta \%=\left\lfloor \frac{O_{C_{i}}-O_{C_{i-1}}}{O_{C_{i-1}}}\times 100\right\rceil$$

In this work we only use percentile differences to assess the area increments and the energy decrements.

### 2.4. Evaluation Environment

We used the Xilinx ISE Design Suite 14.3 for synthesis and configuration of all the architectures described. The designs were described in VHDL and synthesized with *Area Reduction* as design goal and *strategy2* as strategy. All the results provided in this document were obtained after Place and Route (PAR) unless explicitly stated otherwise.

The power estimations reported in this paper correspond to the sum of dynamic and static power. Since for FPGAs the static part tends to outweigh the dynamic power, in some cases the total power might appear somewhat constant.

These estimations were obtained using the Xilinx XPower Analyzer software. In order to obtain a *high* overall confidence level we employed the post-PAR design file (ncd), the physical constraints file (pcf) for the specified FPGA, and a simulation activity file (saif). The latter was obtained using the Xilinx Isim software from a post-PAR simulation; each one of the architectures was simulated using actual data for over 10,000 cycles.

## 3. Energy Reduction in the Literature

Improving the performance of the system is one of the most common approaches for reducing the energy consumption [16,17,18,19,20,29,30,31,32,33,34]. In [35] it is shown that techniques like pipelining and parallelism can be used to reduce the power consumption. If the computations are completed quickly, a moderate rise in the power required (due to increments in the area and switching activity) can be mitigated by the time reduction. In this regard multiple alternatives have been proposed: using low-latency algorithms, proposing low-latency implementations, exploiting algorithm parallelism, and using dedicated processing units. Nonetheless, just as it is inadequate to say that low-area equals *lightweight*, it is also flawed to assume that high-performance equals low-energy. As reviewed in the previous section, the relations between energy and performance are not clear-cut. The other strategies for achieving power reduction consider area minimization [21,22] and exploring area/performance tradeoffs [32,36].

From the perspective of security protocols, it can be concluded that low overheads in the number of packets [22,37,38,39,40] and the number of cryptographic operations [32,38,41,42,43] are key for low-energy PKC. These nodes are characterized by wireless transmissions, which require considerable amounts of energy to be performed, thus it is opportune to use protocols with low packet count requirements. As mentioned, ECC offers the smallest key sizes for comparable security levels. That property holds for all the group elements, thus contributing to reducing the transmissions overhead.

The implementation platform plays a significant role in the design of an ECC system. Using a generic processor would imply selecting prime curves, since commercial ALUs seldom include binary multipliers. On the other hand, a hardware solution would benefit from using binary curves [17].

Selecting the adequate coordinate representation, the group operations, and the field operations used in the ECC system is of paramount importance. For a software-implementation these choices translate into different routines that are executed by the processor, whilst for a hardware-realization these translate into different hardware modules. Processors benefit from shorter routines, from quick calculations, but also from reduced memory accesses [44]. On the other hand, hardware architectures can exploit the arithmetic of binary fields for performing calculations swiftly.

For hardware systems, low latency designs are generally preferred, although implementing faster application-specific modules can result in expensive area investments. How much hardware can be used for improving performance? In the literature, significant interest has been put into concrete points: (a) selecting the optimal field inverter [16,17,18,19,27,30,41,45,46]; (b) determining the adequate digit size for the digit multiplier [17,22,27,38]; (c) designing dedicated squaring modules [16,17,18,25,27,41,45,47].

At circuit level, some works have explored reducing the switching activity of the design by applying clock gating [18,25,26,29,30,38,47,48,49,50], reducing memory accesses [26,30,41,44,46,47], and implementing datapath insulation [38,47].

## 4. Methods

In this section we outline the application and evaluation of different energy-reducing techniques over a low-area *kP* architecture. Throughout the document we use the Binary Edwards Curve BE251 [23] as case study.

We study three architecture-level transformations—field inverter, field multiplier, field squarer—as well as a circuit-level modification—datapath insulation. We study these strategies in the aforementioned order so that the contribution of each technique can be studied in a way in which it benefits the most from previous techniques.

### 4.1. Starting Point: Low-Area kP Architecture

**Figure 1:**
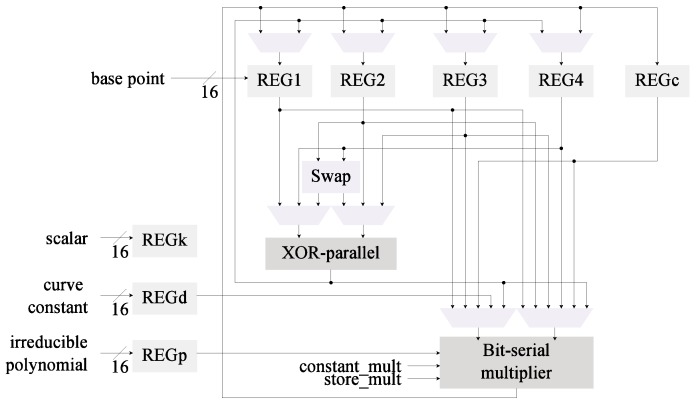
Low-area *kP* architecture, in the following referred to as C0.

In Figure 1 we illustrate the base area-optimized architecture used. This module follows the Montgomery Ladder algorithm with differential addition and doubling for binary Edwards curves in mixed-*w* coordinates as proposed in [24]. One of the main characteristics of this design is that it offers flexibility of the field, curve, base point, and scalar; all the proposed optimizations ought to preserve this property.

The field operations supported by this design are multiplication, addition, and inversion. A bit-serial like multiplier is used to reduce implementation size. Addition is performed by a layer of XOR gates. Field inversion is required to convert the input and output of the system from *w* to projective-*w* coordinates and vice versa. This operation is performed with only multiplications thanks to Fermat’s Little Theorem. The particular inversion algorithm used is Wang’s [51].

In regards to latency, each inversion requires 2m−3*m*-bit multiplications, which amounts to 125,249 cycles when m=251. A step in the Montgomery ladder requires 9×m full multiplications with a latency of 567,009 cycles, *m* short multiplications with a latency of 14,558 cycles, and 3×m additions which take 753 cycles. The architecture requires two inversions and an *m*-bit Montgomery ladder per *kP*, hence the total latency of the design is 832,818 cycles.

While this design performs well in regards to hardware resources, it requires many latency cycles. This has a negative effect on the performance and energy consumption of the system. In the following we review the application of different optimization strategies devised to reduce the energy footprint.

### 4.2. Modification 1: Inversion Algorithm

Field inversion provides a convenient way to perform divisions in finite fields. Such operations are required in point conversion. The scalar multiplication algorithm selected requires two inversions. The Wang inversion algorithm is used in the C0 architecture. Although this method is simple and flexible, more efficient solutions exist.

#### 4.2.1. Fermat’s Little Theorem

Let *q* be a prime number and let *a* be an integer satisfying gcd(a,q)=1 then

(9) $$a^{q-1}\equiv 1\;\mathrm{mod}\;q$$

This conjecture is known as Fermat’s Little Theorem [52]. A simple proof for the theorem is provided in [53]. Consider the product (a)(2a)(3a)…((q−1)a), which can be written as $\left(q-1\right)!a^{q-1}$. The list of terms in the product modulo *q* is a complete list of variables from 1 to q−1, since no two terms in the list are equivalent modulo *q*. From this, the product can also be written as (q−1)!modq. Thus

(10) $$\left(q-1\right)!a^{q-1}\equiv \left(q-1\right)!\;\mathrm{mod}\;q,$$

and (9) is demonstrated.

#### 4.2.2. Divisions on Finite Fields

In 1979, MacWilliams and Sloane demonstrated that every element $a\in F_{p^{m}}$, where $p=2^{n}$, satisfies the identity $a^{p^{m}}=a$. This, together with the demonstration from Wang in 1985 that a non-zero element $a\in F_{p^{m}}$ has a unique multiplicative inverse $a^{-1}$, shows that $a^{-1}=a^{2^{m}-2}$

Then, for all $a\in F_{2^{m}},a\neq 0$, $a^{-1}$ can be computed as

(11) $$a^{-1}=a^{2^{m}-2}=a^{2}\times a^{2^{2}}\times \ldots \times a^{2^{m-1}}$$

according to a generalization of (9). This requires n−2 multiplications and n−1 squarings [54].

Inverses are important in calculating divisions since

(12) $$\frac{a}{b}=c\to ab^{-1}=c$$

Therefore, it is possible to perform divisions through a series of repeated multiplications and squarings.

#### 4.2.3. Wang Inversion

The naïve approach for computing inversions through Fermat’s Little Theorem is denominated Wang Inversion [51]. As presented in Algorithm 1, this operation requires m−2 multiplications and m−1 squarings.

**Algorithm 1** Wang Inversion Method.**Input:**$A\left(x\right)\in F_{2^{m}}$, f(x) the irreducible polynomial of $F_{2^{m}}$ **Output:**
$C\left(x\right)=A^{-1}\left(x\right)\mathrm{mod}f\left(x\right)$ C(x)←A(x) **for**
i=1
**to**
m−2
**do**  $B\left(x\right)\leftarrow C^{2}\left(x\right)\mathrm{mod}f\left(x\right)$  C(x)←A(x)B(x)modf(x) **end for** $C\left(x\right)\leftarrow C^{2}\left(x\right)\mathrm{mod}f\left(x\right)$  **return**
C(x)

Albeit slow, the Wang method of inversion is capable of solving for any A(x) which has an inverse over $F_{2^{m}}$ with *m* of any length. It is also important to note that only two registers are required in this procedure.

(13) $${\left(A^{2^{m-1}-1}\right)}^{2}={\left[\left(A^{2^{2^{k_{t}}}-1}\right){\left(\left(A^{2^{2^{k_{t-1}}}-1}\right)\ldots {\left[\left(A^{2^{2^{k_{2}}}-1}\right){\left(A^{2^{2^{k_{1}}}-1}\right)}^{2^{2^{k_{2}}}}\right]}^{2^{2^{k_{3}}}}\ldots \right)}^{2^{2^{k_{t}}}}\right]}^{2}$$

#### 4.2.4. Itoh-Tsujii Inversion Algorithms

In their work [51], Itoh and Tsujii proposed three field inversion algorithms. The first two of them for inverses over binary fields and the third for inverses over generic fields. The third case, however, relies on subfield inversion.

The first algorithm is applicable in $F_{2^{m}}$ such that $m=2^{r}+1$. It is based on the observation that the exponent $2^{m}-2$ in (11) can be rewritten as $(2^{m-1}-1)\times 2$. Thus if $m=2^{r}+1$, it follows that

(14) $$A^{-1}={\left(A^{2^{2^{r}}-1}\right)}^{2}$$

From this, Algorithm 2 is obtained. This procedure requires $\log_{2}\left(m-1\right)$ multiplications and m−1 squarings.

**Algorithm 2** Itoh-Tsujii Inversion for $F_{2^{m}}$ Where $m=2^{r}+1$.**Input:**$A\left(x\right)\in F_{2^{m}}$ with $m=2^{r}+1$, f(x) the irreducible polynomial of $F_{2^{m}}$ **Output:**
$C\left(x\right)=A^{-1}\left(x\right)\mathrm{mod}f\left(x\right)$ C(x)←A(x) **for**
i=0
**to**
r−1
**do**  B(x)←C(x)  **for**
j=0
**to**
$2^{i}$
**do**   $B\left(x\right)\leftarrow B^{2}\left(x\right)\mathrm{mod}f\left(x\right)$   **end for**  C(x)←C(x)B(x)modf(x) **end for** $C\left(x\right)\leftarrow C^{2}\left(x\right)\mathrm{mod}f\left(x\right)$  **return**
C(x)

Algorithm 2 can be generalized to any value of *m* as proposed in [51]. For this, write m−1 as

(15) $$m-1=\sum_{i=1}^{t}2^{k_{i}}$$

where $k_{1}>k_{2}>\ldots >k_{t}$ is an addition chain. Then, knowing that

(16) $$A^{-1}={\left(A^{2^{m-1}-1}\right)}^{2}$$

and (15), it can be shown that the inverse of *A* can be solved as in (13).

The Itoh-Tsujii inversion for fields of generic length can be computed following two approaches. Note that by calculating $A^{2^{2^{k_{1}}}-1}$, all the previous partial products are also obtained. For posterior use these must be either stored by using additional registers (Algorithm 3) or re-calculated by taking additional operations (Algorithm 4).

**Algorithm 3** Itoh-Tsujii Inversion for Generic Binary Fields Where Extra Storage is Used.**Input:**$A\left(x\right)\in F_{2^{m}}$, $U=u_{0},u_{1},\ldots u_{r-1}$ the binary representation of *m*, f(x) the irreducible polynomial of $F_{2^{m}}$ **Output:**
$C\left(x\right)=A^{-1}\left(x\right)\mathrm{mod}f\left(x\right)$ C(x)←A(x) **for**
i=0
**to**
r−1
**do**  B(x)←C(x)  **for**
j=0
**to**
$2^{i}$
**do**   $B\left(x\right)\leftarrow B^{2}\left(x\right)\mathrm{mod}f\left(x\right)$  **end for**  C(x)←C(x)B(x)modf(x)  $D_{i}\left(x\right)\leftarrow C\left(x\right)$ **end for** **for**
i=r−2
**to** 1 **do**  **if**
$u_{i}=1$
**then**   B(x)←C(x)   **for**
j=0
**to**
$2^{i}$
**do**    $B\left(x\right)\leftarrow B^{2}\left(x\right)\mathrm{mod}f\left(x\right)$   **end for**   $C\left(x\right)\leftarrow C\left(x\right)D_{i}\left(x\right)\mathrm{mod}f\left(x\right)$  **end if** **end for** $C\left(x\right)\leftarrow C^{2}\left(x\right)\mathrm{mod}f\left(x\right)$  **return**
C(x)

**Algorithm 4** Itoh-Tsujii Inversion for Generic Binary Fields Where Additional Cycles are Required.**Input:**$A\left(x\right)\in F_{2^{m}}$, $U=u_{0},u_{1},\ldots u_{r-1}$ the binary representation of *m*, f(x) the irreducible polynomial of $F_{2^{m}}$ **Output:**
$C\left(x\right)=A^{-1}\left(x\right)\mathrm{mod}f\left(x\right)$ C(x)←A(x) **for**
i=0
**to**
r−1
**do**  B(x)←C(x)  **for**
j=0
**to**
$2^{i}$
**do**   $B\left(x\right)\leftarrow B^{2}\left(x\right)\mathrm{mod}f\left(x\right)$  **end for**  C(x)←C(x)B(x)modf(x) **end for** **for**
i=r−2
**to** 1 **do**  **if**
$u_{i}=1$
**then**   B(x)←C(x)   **for**
j=0
**to**
$2^{i}$
**do**    $B\left(x\right)\leftarrow B^{2}\left(x\right)\mathrm{mod}f\left(x\right)$   **end for**   C(x)←A(x)   **for**
j=0
**to**
*i*
**do**    D(x)←C(x)    **for**
k=0
**to**
$2^{j}$
**do**      $D\left(x\right)\leftarrow D^{2}\left(x\right)\mathrm{mod}f\left(x\right)$    **end for**    C(x)←C(x)D(x)modf(x)   **end for**   C(x)←C(x)B(x)modf(x)  **end if** **end for** $C\left(x\right)\leftarrow C^{2}\left(x\right)\mathrm{mod}f\left(x\right)$  **return**
C(x)

These algorithms perform inverses over fields of generic length. The addition chains used are based on the binary representation of the field length. It is possible to compute optimal addition chains, however, this task is difficult to perform on constrained devices given that the field length is variable.

#### 4.2.5. Comparison of the Inversion Methods Reviewed

A summary of the computational and storage costs for the different inversion algorithms reviewed is provided in Table 1. Whereas Table 2 reports the latency and storage estimation of the inversion algorithms for security levels close to 128-bits.

As it can be noted from Table 2, there is an improvement in the number of underlying operations when the Itoh-Tsujii algorithm is implemented over the Wang inversion method. Recall that *kP* requires two field inversions, therefore, reducing the latency of this operation by *x* reduces the latency of the scalar multiplication by 2x.

The alternative in Algorithm 2 only works for fields that satisfy the condition $m=2^{r}+1$ and thus would limit the elliptic curves that can be used if selected. The alternative in Algorithm 3 works for any *m* but its implementation requires increased storage space which would be translated into higher hardware usage—four additional *m*-bit registers if m=251; this increment can be calculated as described in Table 1. Whereas the inversion method in Algorithm 4 does not offer the same latency advantages as the alternatives, it preserves generality without requiring additional hardware resources. Moreover, when the overall *kP* latency is considered and a dedicated squaring module can be added, the performance cost is not as significant (3% difference).

The Itoh-Tsujii inversions exploit the fact that squarings over finite fields are faster than multiplications. To achieve further improvement in the energy consumption of the system, it is necessary to improve the multiplication and squaring modules.

#### 4.2.6. Implementation of the Itoh-Tsujii Inversion

**Figure 2:**
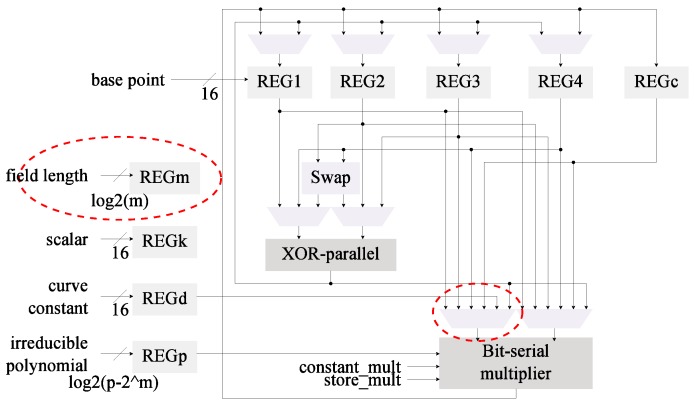
Architecture for *kP* featuring Itoh-Tsujii inversion (C1).

Figure 2 reflects the changes in the architectural design compared to the base architecture in Figure 1. For this second architecture it was necessary to include the field length as an additional input. This value is used to control the iterations in the Itoh-Tsujii inversion algorithm. One of the MUX that feeds the field multiplier input was required to be re-wired as well.

The implementation results for the *kP* architectures (comparing C0 and C1) can be found in Table 3.

From these results it can be noted how the modification of the inversion algorithm offers an average reduction of 7% in the energy consumption for different versions of the *kP* architecture. On the other hand, the hardware usage shows an average increment of 7%. This is consistent with the data in Table 2.

### 4.3. Modification 2: Field Multiplier

In the outlined second strategy, replacing the bit-serial multiplier with a digit multiplier is suggested. The new multiplier should be created with the same ports as the previous one to ease the interconnection; it ought to provide support for fast ×1 operations (which can be used to store data in the registers); and constant multiplications (with reduced length) should also be preserved. The new multiplier also needs to be parameterized in order to function for any digit size and any field length, preserving the generality of the design.

#### 4.3.1. Digit-Based Multiplier

A digit-based multiplier, as presented in Algorithm 5, allows to explore area/latency tradeoffs for different applications. Implementing a digit-based multiplier makes it possible to explore how much hardware can be compromised in order to reduce the cycle count of the architecture. If the design is parameterized then a single architecture can be used for a wide range of applications.

**Algorithm 5** Digit Multiplication in $F_{2^{m}}$ Where *d* is the Digit Size [55].**Input:**$A\left(x\right),B\left(x\right)\in F_{2^{m}}$, $f\left(x\right)=x^{m}+x^{l}+\ldots +1$ the irreducible polynomial of $F_{2^{m}}$ **Output:**
C(x)=A(x)B(x)modf(x) $C\left(x\right)\leftarrow B_{d-1}\left(x\right)A\left(x\right)\;\mathrm{mod}\;f\left(x\right)$ **for**
i=d−2
**to** 0 **do**  $C\left(x\right)\leftarrow x^{l}C\left(x\right)$  $C\left(x\right)\leftarrow B_{i}\left(x\right)A\left(x\right)+C\left(x\right)\;\mathrm{mod}\;f\left(x\right)$ **end for**  **return**
C(x)

The digit multiplier from Algorithm 5 uses an underlying combinatorial multiplier:

(17) $$U\left(x\right)A\left(x\right)\;\mathrm{mod}\;f\left(x\right)=u_{d-1}x^{d-1}A\left(x\right)\;\mathrm{mod}\;f\left(x\right)+\ldots +u_{1}xA\left(x\right)\;\mathrm{mod}\;f\left(x\right)+u_{0}A\left(x\right)\;\mathrm{mod}\;f\left(x\right)$$

The size of this combinatorial multiplier is what determines the hardware cost of the digit multiplier. A combinatorial multiplier can be seen as a matrix of hardware cells where its width is the digit size and its depth is the operand size.

#### 4.3.2. Implementation of the Digit Multiplier

We designed a digit multiplier based on two combinatorial multipliers. The design was synthesized for the xc6slx16 FPGA. At this point, the number of IO ports in the digit multiplier makes the place-and-route process infeasible; some post-synthesis results are provided in Table 4.

**Figure 3:**
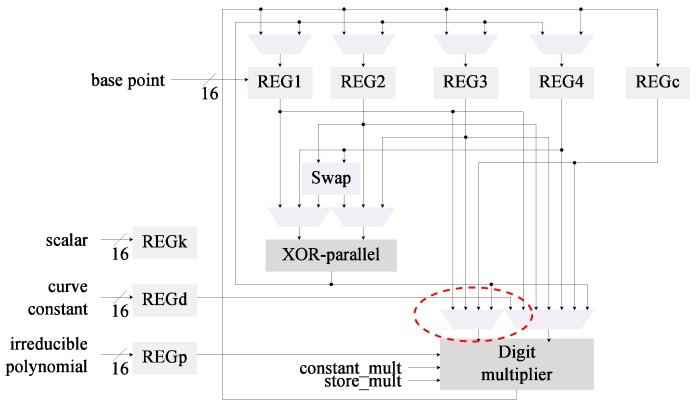
Architecture C2 for *kP*, Wang inversion and a digit-multiplier are used.

The digit multiplier was integrated in the architecture C0 which uses the Wang inversion algorithm to generate a new architecture denominated C2. This aims to determine if the use of Itoh-Tsujii inversion is cost-effective when a dedicated squaring module is not implemented. In this case, as can be seen in Figure 3, the bit-serial multiplier is replaced with the digit multiplier. Only small changes in the input MUX are required.

**Figure 4:**
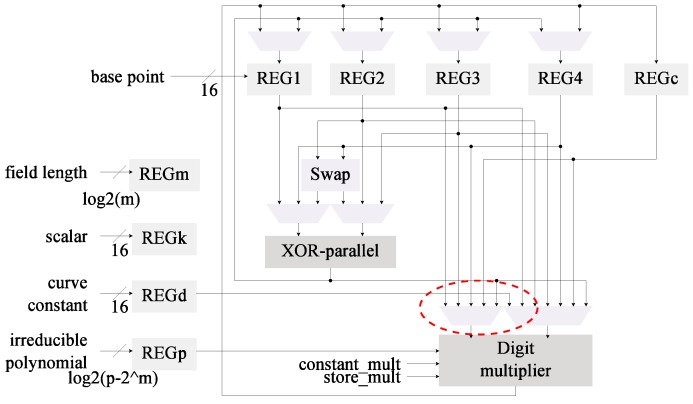
The Itoh-Tsujii inversion is paired with a digit-multiplier on this *kP* architecture (C3).

The multiplier was also merged into architecture C1 to generate the design shown in Figure 4. This architecture now has been modified with the first two proposed optimizations.

The implementation results for C2 and C3, which now use a digit multiplier, can be found in Table 5. Both designs were synthesized for the xc6slx16 FPGA using operational frequencies of 100 KHz and 13.56 MHz. These results are compared against the implementation results for C0 and C1 from Table 3. In this instance we are evaluating the efficiency of the digit multiplier (used in C2 and C3) compared with the bit-serial multiplier (used in C0 and C1).

The use of a digit multiplier enables achievement of a reduction in the energy ranging from 51% to 92% with hardware increments ranging from 6% to 54%. This trend seems to be consistent for both C2 and C3. The main difference between these architectures is that C3 has greater energy reduction for small digit sizes, which implies smaller hardware increments. In the long run (d>16), however, both architectures tend to reach similar energy consumption levels.

### 4.4. Modification 3: Squaring Module

In the base architecture from Figure 1 the squaring operations are realized as multiplications. The selected *kP* algorithm performs four squarings per ladder step (4m). By including a dedicated squaring module the latency can be reduced since squarings are more efficient than multiplications in hardware. The C1 and C3 architectures (Figure 2 and Figure 4, respectively) can also benefit from this modification since the inversion method used (Algorithm 4) relies heavily on squarings. Although the advantages of a dedicated squaring component are evident, the hardware costs must be evaluated in order to assess its efficiency.

A combinatorial design for squarings was selected in order to maximize the latency reduction. Note that using a squaring module can reduce the latency independently of the field multiplier used. For this reason, we study both the alternative where the field multiplication uses a bit-serial approach but that also features dedicated squarings, and the option where the system uses a digit multiplier together with a squaring module.

#### 4.4.1. Field Squarings

A squaring module is a special kind of field multiplier which exploits the fact that both input operands are the same word. The squaring procedure is presented in the following. Following this method, the squaring operation is reduced to a field multiplication of an m−2 bits word by a *d* bits word.

Let the input element *A* be represented as a polynomial A(x):

(18) $$A\left(x\right)=a_{m-1}x^{m-1}+\ldots +a_{2}x^{2}+a_{1}x+a_{0}$$

Thus, the squaring of A(x) in polynomial form is obtained by shifting the polynomial’s coefficients to the left, generating a 2m−1 terms polynomial $A^{2}\left(x\right)$:

(19) $$A^{2}\left(x\right)=a_{m-1}x^{2m-2}+\ldots +a_{2}x^{4}+a_{1}x^{2}+a_{0}$$

The coefficients in $A^{2}$ can be divided in two polynomials $A_{h}\left(x\right)$ and $A_{l}\left(x\right)$, considering that the elements in $A_{h}\left(x\right)$ are shifted m+1 positions to the left:

(20) $$A^{2}\left(x\right)=A_{h}\left(x\right)x^{m+1}+A_{l}\left(x\right)$$

The coefficients in each of the new polynomials are:

(21) $$\begin{array}{c} A_{h}\left(x\right)=a_{m-1}x^{m-3}+\ldots +a_{\frac{m+3}{2}}x^{2}+a_{\frac{m+1}{2}}\\ A_{l}\left(x\right)=a_{\frac{m-1}{2}}x^{m-1}+\ldots +a_{1}x^{2}+a_{0} \end{array}$$

Shifting the elements in $A_{h}\left(x\right)$ can be solved as a multiplication by the element $x^{m+1}$, which can be obtained from the finite field’s irreducible polynomial f(x):

(22) $$\begin{array}{c} f\left(x\right)=x^{m}+x^{d}+\ldots +1\\ x^{m}=x^{d}+\ldots +1\;\mathrm{mod}\;f\left(x\right)\\ x^{m+1}=x^{d+1}+\ldots +x \end{array}$$

The final multiplication can be performed either using a bit-serial multiplier or a combinatorial multiplier. The latter was used for this work.

(23) $$A_{h}\left(x\right)x^{m+1}=A_{h}\left(x\right)\times \left(x^{d+1}+\ldots +x\right)$$

#### 4.4.2. Implementation of the Squaring Module

**Figure 5:**
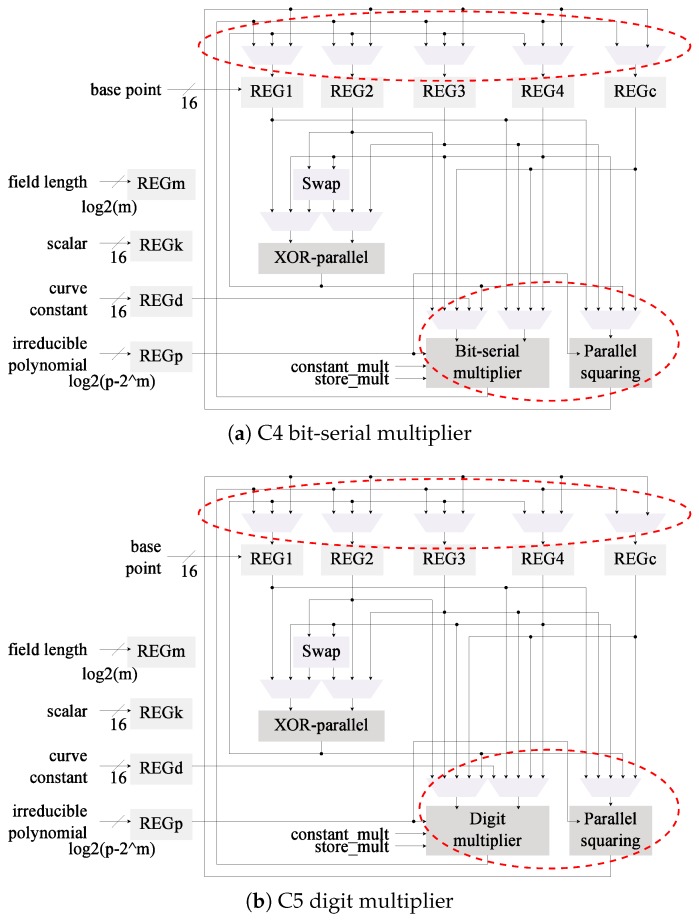
Architectures for *kP* featuring dedicated squaring modules.

The squaring module was included to the architectures that use the Itoh-Tsujii inversion algorithm (C1 and C3) to generate the versions C4 and C5 of the architecture, respectively. The architectural designs of C4 and C5 are illustrated in Figure 5.

In both modules the main difference is the addition of the squaring module, which has an effect on the MUXs at the input of the field multiplier. The MUXs at the input of the data registers were also updated to store the results of the squaring module.

The designs were implemented for the xc6slx16 FPGA using operational frequencies of 100 KHz and 13.56 MHz. Table 6 provides implementation results for the *kP* architectures which include a dedicated squaring module.

In the case where a bit-serial multiplier is used, the energy consumption is halved—compare C4 in Table 6 to C1 in Table 3. The addition of the squaring module enables achieving energy reductions ranging from 77% to 96% if a digit multiplier is used (C5). Comparing C5 to C3—where the reduction ranges from 51% to 92%, see Table 5—the improvement is noticeable. The hardware increment of implementing the squaring module is 20%.

### 4.5. Other Strategies

In a design which contains combinatorial logic and data registers, if these registers are not disconnected from the combinatorial logic spurious calculations will be performed. The switching activity on the combinatorial modules translates into power dissipation, and if the data being processed is not useful then it represents energy being wasted. In order to mitigate the spurious calculations, it is a good design practice to insulate the data registers. This applies both for inputs and outputs. If storing the data is not required, then the register writing must be disabled; if the data in the register is not needed, then it should be masked with zeros.

*Register insulation* is built-in in the proposed designs. As can be noted from Figure 1, Figure 2, Figure 3, Figure 4 and Figure 5 the data registers outputs are always connected to a MUX element. These modules, under all cases default to GND when the output is not required, effectively insulating the data in the registers from reaching any combinatorial module.

We evaluated the impact of removing the register insulation from our designs. Although this strategy has a hardware cost and contributes to reducing the power dissipation, the variation is not significant—13% less energy in the best case and 10% more hardware in the worst case.

### 4.6. Summary

Table 7 provides a summary of all the designs studied in this section.

## 5. Energy Savings in Relation to Area Costs

In this section we describe the design and application of a method to evaluate the efficiency of the optimization techniques that were used to create the *kP* architectures C1–C5. This section is conformed of two parts: first we describe a novel method for quantifying the efficiency of energy optimizations in regards to area cost, then we use this method for comparing our work with other state of the art solutions.

For the analysis provided in this section we consider that the configurations C0, C1, and C4 are equivalent to the configurations C2, C3, and C5 when d=1, respectively.

### 5.1. Novel Metric for Efficiency of Energy Oriented Optimizations in Regards to Area Costs

Since it is complicated to characterize the *efficiency* of an optimization technique in terms of area or energy, we have developed an evaluation metric which can account for both magnitudes.

**Figure 6:**
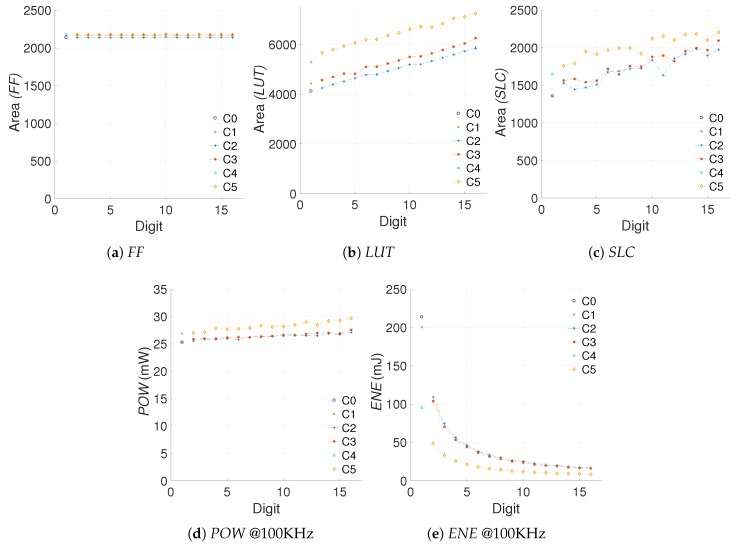
FPGA area, power dissipation, and energy consumption for the different *kP* architectures.

We start from the energy evaluation and area cost of all the hardware implementations. Figure 6 shows the area (*FF, LUT, SLC*), power (*POW*), and energy (*ENE*) results for the different *kP* architectures under study.

In this work we describe four challenges which should be surpassed for an evaluation metric aimed at comparing hardware realizations; these challenges are described in the following.

#### 5.1.1. Selecting the Data

In FPGA implementations it is customary to use *SLCs* as area unit. However, as it can be seen from Figure 6, the *SLC* measurements are prone to outliers. This occurs due to the nature of the PAR process which follows heuristic approaches. In the ideal case the number of *SLC* should be correlated with the number of *FFs* and *LUTs* placed in the design. For example, for the FPGA used in our experiments, each *SLC* contains four *FFs*, four *LUTS*, and some connection logic.

The number of *FFs* required by the *kP* architectures is given by the number of registers allocated. The modifications applied do not modify the number of registers substantially, which is why this value remains almost constant. In contrast, since most of the changes made require combinatorial logic, we can observe that the amount of *LUTs* varies steadily. With this reasoning, we propose to use *LUTs* as area indicator for the configurations evaluated. The *first challenge* for the proposed metric is to define whether the *LUT* results can be used to represent the hardware increment in the design accurately.

In regards to power dissipation and energy consumption, the quiescent component in FPGAs is almost constant and more significant than its dynamic counterpart; this makes it easier to study the energy profile of an architecture.

To measure the area and energy increments we use percentile differences (Δ%). The variation, difference, or increment in the measurement of a particular metric ($O_{C_{i}}$) for the architecture $C_{i}$, with regards to a previous observation ($O_{C_{i-1}}$) for the architecture $C_{i-1}$ can be computed as in (8).

**Figure 7:**
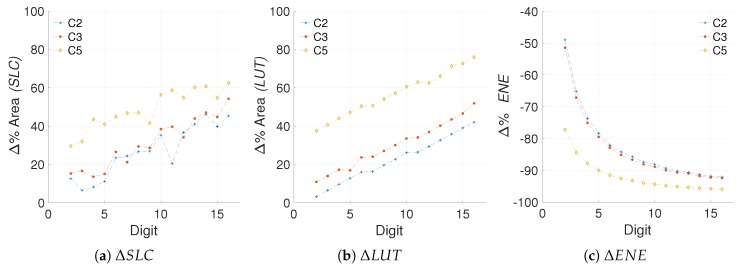
Percentile area and energy increments for architectures C2, C3, and C5 with reference to C0.

Figure 7 shows the area and energy Δs for the different architectures created. It is important to recall that in the proposed scheme a positive difference implies an increment, like in the case of area, and a negative difference implies a decrement, like in the case of energy consumption.

From the results in Figure 7 we can note that in fact, the *LUT* usage is a close match to the *SLC* in regards to perceived hardware cost, with less impact from outliers. The R-square yields a closeness of 74.54%, 98.21% and 16.45% for C2, C3, and C5, respectively. Even though the R-square in the case of C5 is not great, the goodness-of-fit achieved for C2 and C3 hints that the *LUT* measurements can substitute the *SLC* as area units when the number of *FFs* remains constant. This solves the *first challenge* proposed.

#### 5.1.2. Efficiency Metric

Using the *LUT* and *ENE* results we propose the efficiency (*EFF*) metric in (24). What this value conveys is the energy decrement weighted by the area increments associated with the improvement. If the energy savings are high (negative percentages), and the area costs for said improvements are low (in relation to a reference model) then the efficiency metric will yield a high negative result. Results that are less negative imply that the area cost outweighs the energy savings achieved.

(24) $$EFF=\frac{\Delta ENE}{\Delta LUT}$$

**Figure 8:**
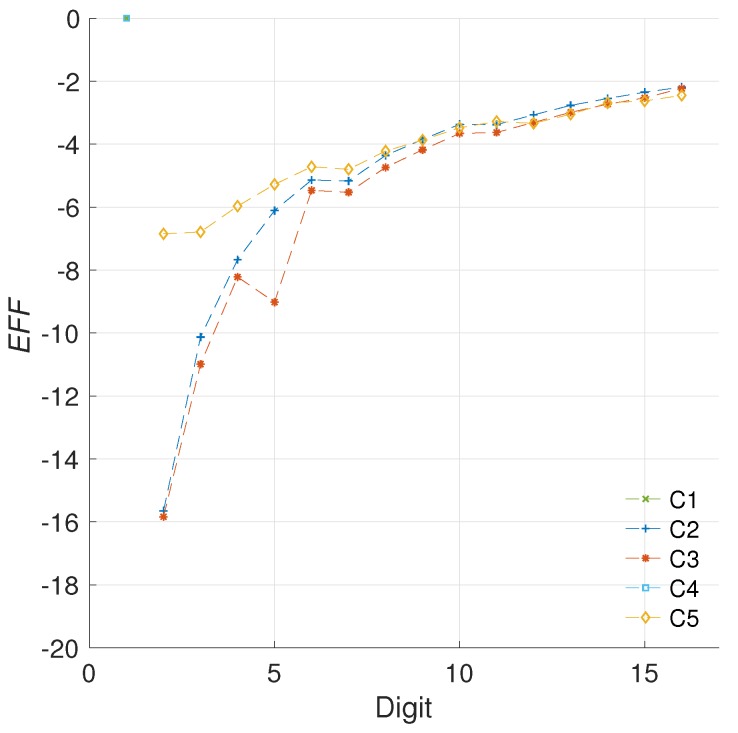
Evaluation of the efficiency metric for the different *kP* configurations.

Figure 8 presents the evaluation of the efficiency metric for the different architectures created.

#### 5.1.3. Sensitivity to Frequency Variations

For the results in Figure 8 we used an operational frequency of 100 KHz. However, how does the operational frequency affect the proposed evaluation metric? This is the *second challenge* for our metric. This question is important for comparing our results with proposals from the literature. It is evident that not all the works would use the same operational frequency. Furthermore, given the relevance of this magnitude in the energy consumption of a design, it is clear that any evaluation metric should account for frequency variations.

**Figure 9:**
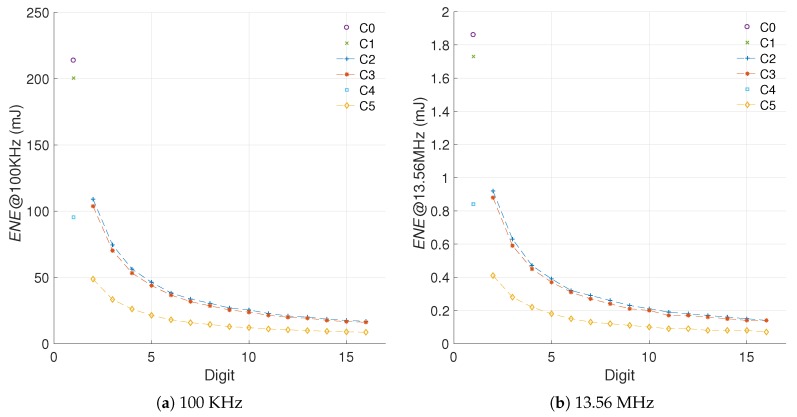
Energy consumption of the *kP* architectures at different operational frequencies.

Figure 9 illustrates the differences in the energy consumption for the *kP* architectures under evaluation as a result of changing the operational frequency.

As it can be noted, even though the measured values vary by two orders of magnitude, the consumption models are similar. In fact, both energy measurements can be used for computing the energy increment of each architecture, and later the efficiency evaluation using both operational frequencies.

**Figure 10:**
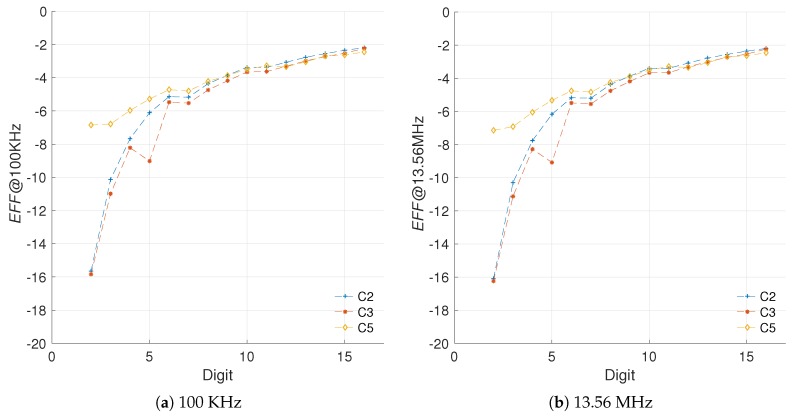
Evaluation of the efficiency metric for the different *kP* configurations using two operational frequencies.

The evaluation of the efficiency metric for two operational frequencies is provided in Figure 10. The results demonstrate that the proposed metric can account for variations in the operational frequency of the implementation. In these results, the R-square for the configurations C2, C3, and C5 indicates goodness values of 99.87%, 99.91%, and 99.62%, respectively. This answers the *second challenge* proposed, since the metric proposed appears to be able to isolate the strong influence that the frequency has on the static power consumption, while highlighting the improvements achieved by the architecture modifications on the dynamic power consumption.

#### 5.1.4. Sensitivity to Different Curve Sizes

How does the curve length influence the results? This is considered the *third challenge* proposed. In this work we use the elliptic curve BE251 as case study. However, when comparing our work with the literature, it is noteworthy that most existing lightweight proposals of elliptic curve systems target security levels of at most 80 bits. Since our work targets security levels close to 128 bits, the necessity of accounting for the difference in field length is clear.

**Figure 11:**
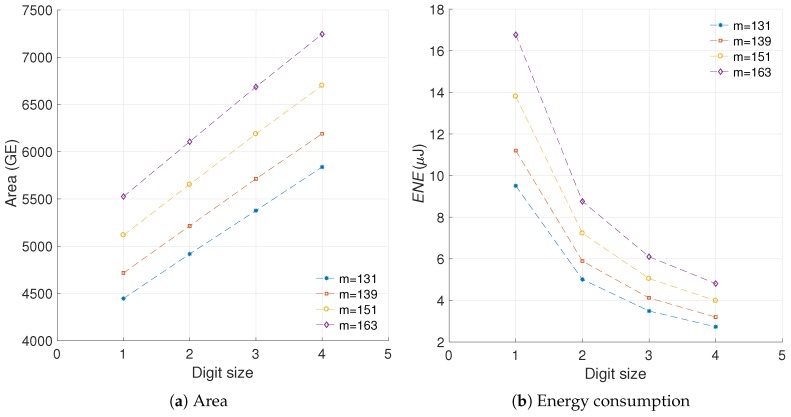
Implementation results for the architectures in [22].

**Figure 12:**
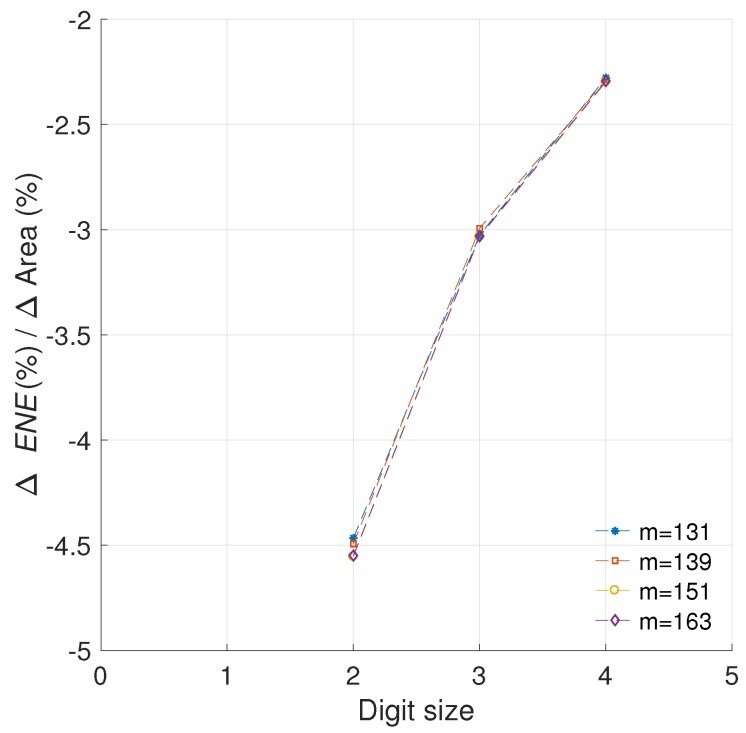
Evaluation of the efficiency metric for the results from [22].

We have used the results provided in [22] to evaluate the sensitivity of the proposed metric to differences in the curve length. The relevance of that work is that the authors present implementation results for a scalar multiplication architecture using generic elliptic curves of varying length. We took their area and energy results and utilized them to evaluate our metric. Figure 11 illustrates the area and energy results from [22] for various curve lengths. Figure 12 presents the evaluation of the proposed metric using the results from [22]. In this case the area increments are measured in GEs and the energy increments in μJ.

As it can be noted from Figure 12, our metric yields similar calculations for the different experiments which use varying curve lengths. These figures generate R-square evaluations of 99.99%, 99.99%, and 99.99%, which implies a close match in the values. Based on this experiment, we can conclude that the proposed metric is not sensitive to variations in the curve length, which solves the *third challenge* presented. This is a significant result as it implies that in comparing our results with the state of the art it is not necessary to account for variations in the curve length.

#### 5.1.5. Sensitivity to the Implementation Technology

As evidenced in the previous point, not all works in the literature target FPGA technology. Some of them, as in the case of [22], have been developed for ASIC. Even though our metric can be applied to both scenarios without problems, it is necessary to determine if changing the implementation technology can impact the results of the proposed metric for the same architecture. However, our work focuses on FPGA technology and in the literature we have not identified any work which allows carrying out this experiment. As of now, we consider this fourth question as an *open challenge*.

### 5.2. Applying the Proposed Metric for Comparing Our Work with the State of the Art

All of the reviewed works which propose low-power or low-energy *kP* architectures use digit multipliers. This is understandable, given how a digit multiplier allows for significant improvements in the reduction of the energy consumption with relative low hardware costs.

The metric proposed is particularly useful for comparing such works. For starters, the ability of synthesizing a design for varying digit sizes allows flexibility of the application. Some scopes might be able to accommodate greater hardware strains in order to achieve improved performance, whereas others can have stricter area bounds. Therefore, an architecture of this type cannot be evaluated solely on the efficiency for a particular digit size. The curves derived from the evaluation of the efficiency metric proposed, as a function of the digit size in architectures with digit multipliers, make it possible to use the area under the curve as an objective quantifier of efficiency. To this end different problems need to be addressed.

First, since in this case of comparison we refer to the efficiency of a particular architecture which uses a digit multiplier, each series shall use as reference the instance of the implementation where d=1. In this scenario we aim at quantifying the efficiency of an individual architecture. The relative percentile increments can be computed as in (25).

(25) $$\Delta_{r}\%=\left\lfloor \frac{O_{C_{i,d}}-O_{C_{i,1}}}{O_{C_{i,1}}}\times 100\right\rceil$$

Second, to use the area under the curve as quantifier it is necessary that the evaluation bounds are coincident for each configuration. That is, that all the designs evaluated provide implementation results for the same digit interval. The case where d=1 is mandatory since it is used as reference, but as upper bound we can define any d=n.

Once the evaluation boundaries have been defined, we note that the majority of works in the literature do not provide results for continuous intervals of the digit space. For instance, the works in [17] and [25] only provide implementation results for the cases where d∈{1,15} and d∈{1,16}, respectively. A solution for this problem is to use interpolation models in order to obtain the missing data.

#### 5.2.1. Modeling the Data

The area and energy increments are the source for computing the efficiency of a design. These increments are calculated from the raw data of hardware resources and energy consumption. The former can be modeled using a polynomial fit of first degree of the form $y=\alpha_{1}d+\alpha_{2}$ while the latter can be adjusted to an exponential model of the form $y=\alpha_{3}d^{\alpha_{4}}$ where $\alpha_{i}\in R$ are constants for the model of each configuration and d∈Z is the digit size. The model proposed for the efficiency metric is presented in (26).

(26) $${EFF}_{m}=\frac{\Delta_{r}\%\left(\alpha_{3}d^{\alpha_{4}}\right)}{\Delta_{r}\%\left(\alpha_{1}d+\alpha_{2}\right)}$$

The use of a mathematical model over the raw data has the additional advantage that the effects of outliers are mitigated. This is practical since some works from the literature that target FPGAs do not provide *LUT* results [25] or do provide them but the variance in the flip flop count is significant [17].

**Figure 13:**
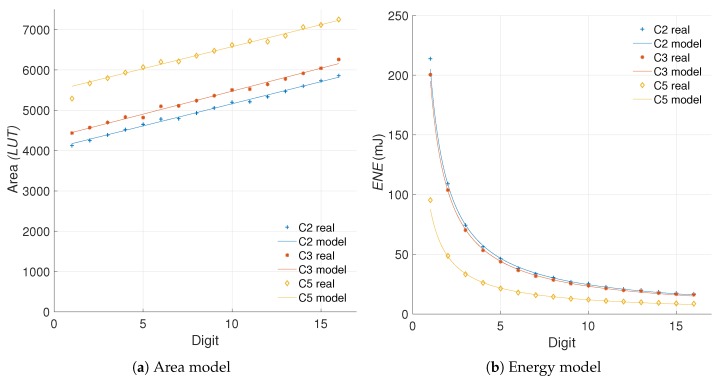
Curve fitting for the hardware usage and energy consumption of architectures C2, C3, and C5.

**Figure 14:**
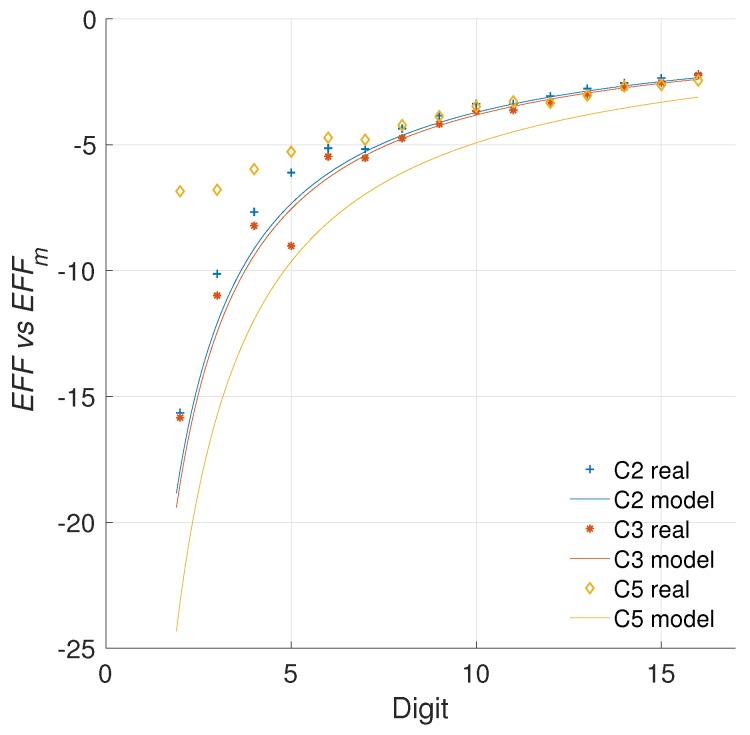
Evaluation of the efficiency metric for the C2, C3, and C5 configurations based on model data (${EFF}_{m}$), compared to the evaluation based on real data (*EFF*).

In Figure 13 we show the models obtained for the area and energy results from our C2, C3, and C5 architectures. Consequently, Figure 14 presents the evaluation of the efficiency metric applied over these data. It is possible to observe the precision obtained in the final model, which produces R-square evaluations of 93.12%, 93.66% for C2 and C3, respectively.

As can be observed in Figure 13, the area in *LUTs* recorded for the configuration C5 where d=1 is an outlier. When this anomalous reference point is used for evaluating (24), the results are skewed. Modeling the data prevents obtaining erroneous results by removing the outliers. This is the reason for the significant variation exhibited between C5 and ${C5}_{m}$.

With the updated analysis we can note that the most cost effective solution provided in this work, in regards to preserving the implementation area while reducing the energy profile, is the architecture C5. This design consistently outperforms the other configurations for any digit size.

#### 5.2.2. Quantifying the Efficiency

The data in Figure 14 can be used to obtain the area under the curve for each configuration using a trapezoidal rule as shown in Equation (27).

(27) $${EFF}_{A}=\frac{1}{2}\sum_{d=2}^{n}\left[\frac{\Delta_{r}\%\left(\alpha_{3}d^{\alpha_{4}}\right)}{\Delta_{r}\%\left(\alpha_{1}d+\alpha_{2}\right)}+\frac{\Delta_{r}\%\left(\alpha_{3}{\left(d+1\right)}^{\alpha_{4}}\right)}{\Delta_{r}\%\left(\alpha_{1}\left(d+1\right)+\alpha_{2}\right)}\right]\Delta_{d}\;\mathrm{where}\;\alpha_{i}\in R\;\mathrm{and}\;d\in Z$$

For this evaluation we shall define n=15 and $\Delta_{d}=1$ since d∈Z. From this, the configurations C2, C3, and C5 obtain efficiency scores of −77.59, −80.16, and −97.5, respectively. In this evaluation, the configuration C5 is the one that achieves the greater energy reduction per area cost overall.

#### 5.2.3. Comparison with the Literature

Table 8 provides implementation results from works in the literature that are defined as “low power” or “low energy” by their authors. Using these data we have adjusted coefficients for the model of the area and energy measurements from each work. These models are used for evaluating the efficiency metric and to obtain the respective efficiency score for each design. In 77% of the non-trivial models we achieved R-square evaluations above 99%, which implies that the provided results are accurate. Table 9 provides the coefficients obtained for the model of each configuration, according to the formula in Equation (26).

**Figure 15:**
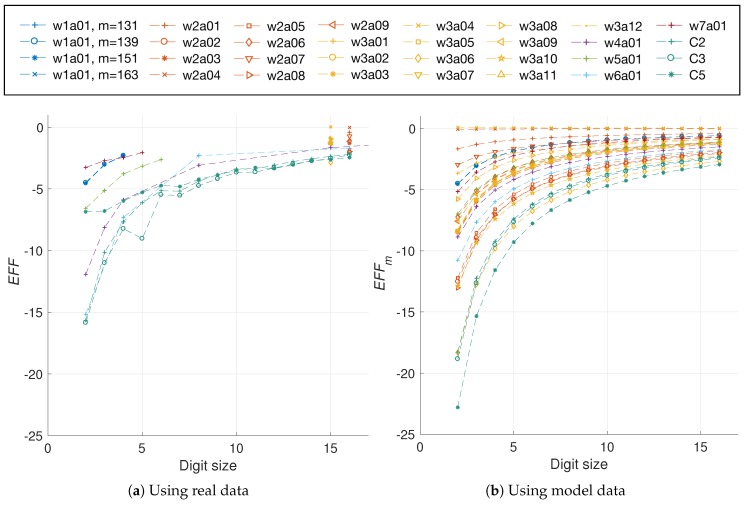
Evaluation of the efficiency metric for the different works in the literature, ours included.

Figure 15 illustrates the evaluation of the efficiency metric for the different works in the state of the art.

**Figure 16:**
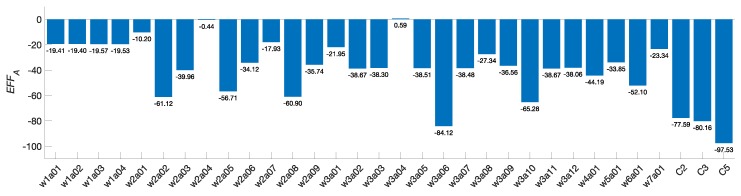
Efficiency scores for the different architectures in the literature. Values that are more negative represent greater energy savings overall.

Finally, the efficiency scores for each configuration evaluated are reported in Figure 16.

### 5.3. Limitations of the Proposed Method

The proposed method is sensitive to data outliers. Since the results are provided as percentages, when the measurements are small, area or energy variations can skew the results. This is solved by using models to adjust the data.

Conditions that do not adjust to the models proposed also lead to unexpected results. If the energy consumption is not reduced or the hardware requirements are not increased, the sign of the results will flop and produce spurious evaluations of (27). While that might have some use, for the purposes intended in this article such results are undesired.

## 6. Conclusions

In this paper we have studied the reduction of energy consumption in six different scalar multiplication architectures. Starting from a base low-area design, we have improved it following energy and power reducing strategies. The result of this process is a comprehensive set of designs that have gradual optimization levels, and thus exhibit from moderate to increased area/energy tradeoffs. These scalar multiplication modules can be used in key establishment systems with low-area requirements and low-energy consumption.

The novel metric proposed can be applied in studying the impact of any modification to a reference architecture, implemented in hardware. In a sense, it represents the energy costs, weighted by the associated hardware costs. The main goal for using this indicator is to demonstrate the effectiveness of any energy-related improvement in a platform with hardware constraints. We have shown that this metric is capable of accounting for differences in the area units, the operational frequency, and the field size; we also provided a way to reduce its sensitivity to unavailable data and outliers. For these reasons we believe that it is adequate for comparing works implemented under heterogeneous conditions.

From the proposed architectures, the configuration C5 exhibits the greatest efficiency. This design employs a digit-multiplier, the Itoh-Tsujii inversion algorithm, and a dedicated squaring module; and also implements datapath insulation. Compared against the state of the art, this configuration turned out to be 13.33% more efficient than the closest work. In terms of efficiency, our proposal represents a good candidate for implementation in environments with area and energy constraints such as IoT devices.

## Figures and Tables

**Table 1:** Inversion algorithms cost over binary fields of variable length. Let v=HW(m−1) and $u_{1}\ldots u_{i}$ the binary representation of *m*, where HW(w) represents the Hamming weight of *w*.

Inv.	Field	Multiplications	Squarings	Storage Bits
Algorithm 1	$F_{2^{m}},m\in Z$	m−2	m−1	2×m
Algorithm 2	$F_{2^{m}},m=2^{r}+1\in Z$	$\log_{2}\left(m-1\right)$	m−1	2×m
Algorithm 3	$F_{2^{m}},m\in Z$	$\lfloor \log_{2}\left(m-1\right)\rfloor +v-1$	m−1	(2+v−1)×m
Algorithm 4	$F_{2^{m}},m\in Z$	$\left\lfloor \log_{2}\left(m-1\right)\right\rfloor +v-1+\sum_{i=1}^{r-2}\left(u_{i}\times i\right)$	$m-1+\sum_{i=1}^{r-2}\left(u_{i}\times \sum_{j=1}^{i}2^{j-1}\right)$	3×m

**Table 2:** Latency costs for inversion algorithms and *kP* over binary fields of approximately 128-bit security. We evaluate m=251 which corresponds with the curve used (BE251) and m=257 which has the form $m=2^{8}+1$ to showcase the best and the average complexities for Itoh-Tsujii inversions.

Inv.	*m*	M	S	MEM	*LAT* (Cycles) ^*a*^		Improvement ^*a*^				*LAT* (Cycles) ^*b*^		Improvement ^*b*^			
				(bits)	Inv.	*kP*	Inv. ΔLAT	Inv. Δ%	*kP* ΔLAT	*kP*Δ%	Inv.	*kP*	Inv. ΔLAT	Inv. Δ%	*kP* ΔLAT	*kP*Δ%
Algorithm 1	251	249	250	502	125,249	832,818	-	-	-	-	62,749	456,818	-	-	-	-
	257	255	256	514	131,327	872,772	-	-	-	-	65,791	478,532	-	-	-	-
Algorithm 2	257	8	256	514	67,848	745,814	−63,479	−48	−126,958	−15	2312	351,574	−60,437	−92	−126,958	−27
Algorithm 3	251	12	250	1757	65,762	713,844	−59,487	−47	−118,974	−14	3262	337,844	−59,487	−95	−118,974	−26
	257	8	256	1799	67,848	745,814	−63,479	−48	−126,958	−15	2312	351,574	−63,479	−96	−126,958	−27
Algorithm 4	251	31	367	753	99,898	782,116	−25,351	−20	−50,702	−6	8148	347,616	−54,601	−87	−109,202	−24
	257	8	256	771	67,848	745,814	−63,479	−48	−126,958	−15	2312	351,574	−63,479	−96	−126,958	−27

^*a*^ Field multiplications (M) and squarings (S) are performed using a bit-serial multiplier. ^*b*^ Multiplications are performed using a bit-serial multiplier and squarings are considered to take 1 cycle.

**Table 3:** Implementation results for C0 and C1 at frequencies of $f_{1}$ = 100 KHz and $f_{2}$ = 13.56 MHz in the xc6slx16 FPGA.

Arch.	*m*	*FF*	*LUT*	*SLC*		*Fmax* (MHz)	*LAT*		*t* (ms)		*POW* (mW)		*ENE* (mJ)			
				#	Δ%		(Cycles)	Δ%	$f_{1}$	$f_{2}$	$f_{1}$	$f_{2}$	$f_{1}$	Δ%	$f_{2}$	Δ%
C0	127	1140	2220	633	-	122	223,024	-	2230.24	16.45	23.59	26.89	52.61	-	0.44	-
	163	1432	2755	868	-	119	362,530	-	3625.30	26.74	24.08	27.43	87.30	-	0.73	-
	233	1994	3877	1224	-	102	730,248	-	7302.48	53.85	25.38	29.87	185.34	-	1.61	-
	251	2138	4122	1357	-	109	845,395	-	8453.95	62.34	25.28	29.83	213.72	-	1.86	-
C1	127	1168	2370	716	13	83	212,096	−5	2120.96	15.64	23.66	27.04	50.18	−5	0.42	−4
	163	1462	2981	945	9	99	324,596	−10	3245.96	23.94	24.13	27.50	78.33	−10	0.66	−10
	233	2024	4173	1311	7	97	680,096	−7	6800.96	50.15	25.15	29.65	171.04	−8	1.49	−8
	251	2168	4435	1352	0	99	793,978	−6	7939.78	58.55	25.24	29.61	200.40	−6	1.73	−7

**Table 4:** Preliminary results for the digit-based multiplier on $F_{2^{251}}$.

Digit	*FF*	*LUT*	*LAT* (Cycles)
2	510	786	129
4	507	1052	66
8	506	1643	35
16	497	2662	19

**Table 5:** Implementation results for C2 and C3 at frequencies of $f_{1}$ = 100 KHz and $f_{2}$ = 13.56 MHz, and variable multiplier digit size in the xc6slx16 FPGA. The Δ% for C2 and C3 were computed in relation to C0 and C1, respectively.

Arch.	Digit	*FF*	*LUT*	*SLC*		*Fmax* (MHz)	*LAT*		*t* (ms)		*POW* (mW)		*ENE* (mJ)			
				#	Δ%		(Cycles)	Δ%	$f_{1}$	$f_{2}$	$f_{1}$	$f_{2}$	$f_{1}$	Δ%	$f_{2}$	Δ%
C2	2	2138	4251	1527	13	88	426,980	−49	4269.80	31.49	25.54	29.30	109.05	−49	0.92	−51
	3	2138	4387	1445	6	98	287,843	−66	2878.43	21.23	25.87	29.57	74.46	−65	0.63	−66
	4	2137	4518	1468	8	93	218,149	−74	2181.49	16.09	25.80	29.37	56.28	−74	0.47	−75
	5	2140	4650	1509	11	88	178,288	−79	1782.88	13.15	25.98	29.59	46.32	−78	0.39	−79
	6	2137	4780	1675	23	63	148,455	−82	1484.55	10.95	25.76	29.32	38.24	−82	0.32	−83
	7	2137	4793	1688	24	91	128,650	−85	1286.50	9.49	26.18	30.06	33.68	−84	0.29	−84
	8	2140	4932	1718	27	88	115,363	−86	1153.63	8.51	26.44	30.28	30.5	−86	0.26	−86
	9	2136	5057	1722	27	85	102,076	−88	1020.76	7.53	26.35	30.25	26.9	−87	0.23	−88
	10	2144	5198	1836	35	86	95,307	−89	953.07	7.03	26.70	30.50	25.45	−88	0.21	−89
	11	2137	5210	1634	20	90	85,530	−90	855.30	6.31	26.63	30.45	22.78	−89	0.19	−90
	12	2136	5334	1855	37	81	78,761	−91	787.61	5.81	26.50	30.33	20.87	−90	0.18	−90
	13	2144	5469	1914	41	80	75,502	−91	755.02	5.57	26.49	30.36	20	−91	0.17	−91
	14	2136	5598	1983	46	78	68,984	−92	689.84	5.09	26.85	30.75	18.52	−91	0.16	−91
	15	2139	5731	1896	40	79	65,474	−92	654.74	4.83	26.72	30.40	17.49	−92	0.15	−92
	16	2139	5856	1972	45	76	62,215	−93	622.15	4.59	27.11	30.93	16.87	−92	0.14	−92
C3	2	2168	4570	1564	15	84	401,075	−53	4010.75	29.58	25.87	29.64	103.76	−51	0.88	−53
	3	2168	4697	1583	17	77	270,464	−68	2704.64	19.95	25.97	29.75	70.24	−67	0.59	−68
	4	2167	4831	1541	14	82	205,033	−76	2050.33	15.12	25.98	29.86	53.27	−75	0.45	−76
	5	2170	4819	1561	15	88	167,608	−80	1676.08	12.36	26.16	29.98	43.85	−79	0.37	−80
	6	2167	5098	1717	27	81	139,602	−83	1396.02	10.30	26.24	30.11	36.63	−83	0.31	−83
	7	2167	5110	1644	21	79	121,015	−86	1210.15	8.92	26.23	30.04	31.74	−85	0.27	−85
	8	2170	5237	1756	29	78	108,540	−87	1085.40	8.00	26.29	29.99	28.54	−87	0.24	−87
	9	2166	5362	1746	29	74	96,065	−89	960.65	7.08	26.44	30.17	25.4	−88	0.21	−89
	10	2174	5503	1878	38	72	89,702	−89	897.02	6.62	26.51	30.23	23.78	−89	0.2	−89
	11	2167	5526	1895	40	73	80,534	−90	805.34	5.94	26.57	29.19	21.4	−90	0.17	−91
	12	2166	5643	1820	34	69	74,171	−91	741.71	5.47	26.82	30.78	19.89	−91	0.17	−91
	13	2174	5778	1953	44	72	71,115	−92	711.15	5.24	26.99	30.75	19.19	−91	0.16	−91
	14	2166	5915	1995	47	71	65,003	−92	650.03	4.79	27.03	30.75	17.57	−92	0.15	−92
	15	2169	6042	1965	45	71	61,696	−93	616.96	4.55	26.92	30.72	16.61	−92	0.14	−92
	16	2169	6260	2093	54	68	58,640	−93	586.40	4.32	27.56	31.36	16.16	−92	0.14	−92

**Table 6:** Implementation results for C4 and C5 at frequencies of $f_{1}$ = 100 KHz and $f_{2}$ = 13.56 MHz in the xc6slx16 FPGA. The Δ% for C4 and C5 were computed in relation to C1 and C3, respectively.

Arch.	Digit	*FF*	*LUT*	*SLC*		*Fmax* (MHz)	*LAT*		*t* (ms)		*POW* (mW)		*ENE* (mJ)			
				#	Δ%		(Cycles)	Δ%	$f_{1}$	$f_{2}$	$f_{1}$	$f_{2}$	$f_{1}$	Δ%	$f_{2}$	Δ%
C4	1	2176	5290	1651	22	88	354,264	−55	3542.64	26.13	26.92	32.11	95.37	−52	0.84	−52
C5	2	2176	5668	1758	30	85	180,349	−79	1803.49	13.30	27.01	30.88	48.71	−77	0.41	−78
	3	2176	5797	1790	32	84	122,734	−85	1227.34	9.05	27.14	31.22	33.31	−84	0.28	−85
	4	2175	5934	1948	44	84	93,801	−89	938.01	6.92	27.84	31.99	26.11	−88	0.22	−88
	5	2178	6068	1912	41	82	77,232	−91	772.32	5.70	27.68	31.83	21.38	−90	0.18	−90
	6	2175	6199	1966	45	85	64,868	−92	648.68	4.78	27.74	31.80	17.99	−92	0.15	−92
	7	2175	6210	1992	47	88	56,709	−93	567.09	4.18	27.92	32.02	15.83	−93	0.13	−93
	8	2178	6353	1995	47	89	51,186	−94	511.86	3.77	28.31	32.29	14.49	−93	0.12	−94
	9	2174	6474	1920	41	82	45,663	−95	456.63	3.37	28.10	32.12	12.83	−94	0.11	−94
	10	2182	6618	2122	56	77	42,776	−95	427.76	3.15	28.20	32.33	12.06	−94	0.10	−95
	11	2175	6716	2153	59	75	38,822	−95	388.22	2.86	28.50	32.96	11.06	−95	0.09	−95
	12	2174	6702	2101	55	75	35,935	−96	359.35	2.65	29.02	33.37	10.43	−95	0.09	−95
	13	2182	6847	2175	60	72	34,617	−96	346.17	2.55	28.46	32.61	9.85	−95	0.08	−96
	14	2174	7062	2182	61	73	31,981	−96	319.81	2.36	29.20	33.87	9.34	−96	0.08	−96
	15	2177	7117	2099	55	70	30,412	−96	304.12	2.24	29.27	33.45	8.90	−96	0.08	−96
	16	2177	7251	2205	62	69	29,094	−97	290.94	2.15	29.67	33.96	8.63	−96	0.07	−96

**Table 7:** Details of the six different *kP* architectures created, highlighting the approach used for performing field operations.

Conf.	Multiplication	Inversion	Addition	Squaring
C0	Bit-serial	Wang	Combinatorial	Not supported
C1	Bit-serial	Itoh-Tsujii	Combinatorial	Not supported
C2	Digit-serial	Wang	Combinatorial	Not supported
C3	Digit-serial	Itoh-Tsujii	Combinatorial	Not supported
C4	Bit-serial	Itoh-Tsujii	Combinatorial	Combinatorial
C5	Digit-serial	Itoh-Tsujii	Combinatorial	Combinatorial

**Table 8:** Implementation results for different low-power or low-area *kP* architectures from the literature.

Year	Ref.	*m*	Curve	Platform	Label	Digit	*FF*	*LUT*	*SLC*	GE	Storage	*LAT* (Cycles)	Freq. (MHz)	*t* (ms)	*POW* (μW)	*ENE* (μJ)
2006	[22]	131	B131	0.13 μm	w1a01	1	x	x	x	4446	5·m bits	226,330	0.50	452.66	21.00	9.51
						2	x	x	x	4917	5·m bits	116,480	0.50	232.96	21.50	5.01
						3	x	x	x	5376	5·m bits	79,300	0.50	158.60	22.00	3.49
						4	x	x	x	5837	5·m bits	60,710	0.50	121.42	22.50	2.73
		139	B139			1	x	x	x	4716	5·m bits	254,610	0.50	509.22	22.00	11.20
						2	x	x	x	5214	5·m bits	130,824	0.50	261.65	22.50	5.89
						3	x	x	x	5712	5·m bits	89,562	0.50	179.12	23.00	4.12
						4	x	x	x	6189	5·m bits	68,034	0.50	136.07	23.50	3.20
		151	B151			1	x	x	x	5117	5·m bits	300,150	0.50	600.30	23.00	13.81
						2	x	x	x	5652	5·m bits	153,900	0.50	307.80	23.50	7.23
						3	x	x	x	6187	5·m bits	105,150	0.50	210.30	24.00	5.05
						4	x	x	x	6700	5·m bits	79,800	0.50	159.60	25.00	3.99
		163	B163			1	x	x	x	5525	5·m bits	349,434	0.50	698.87	24.00	16.77
						2	x	x	x	6105	5·m bits	178,848	0.50	357.70	24.50	8.76
						3	x	x	x	6685	5·m bits	121,986	0.50	243.97	25.00	6.10
						4	x	x	x	7243	5·m bits	92,502	0.50	185.00	26.00	4.81
2007	[25]	163	B163	xc3s1000l	w2a01	1	-	-	2541	x	RAM/ROM/Pro	130,141	80.00	1.63	207,328.39	339.62
						16	-	-	3721	x	RAM/ROM/Pro	92,958	80.00	1.16	236,085.34	274.87
					w2a02	1	-	-	2692	x	RAM/ROM/Pro	287,324	80.00	3.59	171,614.10	610.82
						16	-	-	3728	x	RAM/ROM/Pro	40,564	80.00	0.51	252,319.11	129.49
					w2a03	1	-	-	1551	x	RAM/ROM/Pro	287,324	80.00	3.59	155,380.33	549.74
						16	-	-	2556	x	RAM/ROM/Pro	40,564	80.00	0.51	173,933.21	87.96
					w2a04	1	-	-	2541	x	RAM/ROM/Pro	112,677	80.00	1.41	208,719.85	287.09
						16	-	-	3728	x	RAM/ROM/Pro	112,677	80.00	1.41	205,009.28	284.64
					w2a05	1	-	-	2541	x	RAM/ROM/Pro	174,648	80.00	2.18	217,996.29	472.77
						16	-	-	3728	x	RAM/ROM/Pro	25,353	80.00	0.32	224,953.62	69.63
					w2a06	1	-	-	1543	x	RAM/ROM/Pro	17,4648	80.00	2.18	153,525.05	333.51
						16	-	-	2707	x	RAM/ROM/Pro	25,353	80.00	0.32	179,035.25	54.97
					w2a07	1	-	-	3033	x	RAM/ROM/Pro	116,057	80.00	1.45	222,634.51	322.51
						16	-	-	4061	x	RAM/ROM/Pro	82,817	80.00	1.04	233,766.23	244.33
					w2a08	1	-	-	2624	x	RAM/ROM/Pro	238,874	80.00	2.99	212,430.43	631.59
						16	-	-	3751	x	RAM/ROM/Pro	33,803	80.00	0.42	226,345.08	97.73
					w2a09	1	-	-	1641	x	RAM/ROM/Pro	238,874	80.00	2.99	157,235.62	471.55
						16	-	-	2821	x	RAM/ROM/Pro	33,803	80.00	0.42	175,324.68	76.96
2009	[17]	163	B163	xc3s500e	w3a01	1	3323	3249	2873	x	7 BRAM	126,836	10.00	12.68	76,730.00	973.18
						15	3337	5238	3738	x	7 BRAM	89,976	10.00	9.00	78,500.00	706.26
					w3a02	1	2005	1768	1551	x	8 BRAM	281,024	10.00	28.10	73,680.00	2070.63
						15	2019	3748	2575	x	8 BRAM	33,720	10.00	3.37	84,650.00	285.45
					w3a03	1	2005	1768	1551	x	8 BRAM	226,110	10.00	22.61	73,710.00	1666.58
						15	2019	3748	2575	x	8 BRAM	28,054	10.00	2.81	84,920.00	238.23
					w3a04	1	3323	3249	2873	x	7 BRAM	111,188	10.00	11.12	77,230.00	858.70
						15	3337	5238	3783	x	7 BRAM	110,884	10.00	11.09	78,080.00	865.82
					w3a05	1	2005	1768	1551	x	8 BRAM	171,796	10.00	17.18	73,810.00	1267.93
						15	2019	3748	2575	x	8 BRAM	21,164	10.00	2.12	83,940.00	177.65
					w3a06	1	3323	3249	2873	x	7 BRAM	170,214	10.00	17.02	75,700.00	1288.45
						15	3337	5238	3738	x	7 BRAM	21,181	10.00	2.12	85,890.00	181.93
					w3a07	1	2005	1768	1551	x	8 BRAM	172,124	10.00	17.21	73,640.00	1267.59
						15	2019	3748	2575	x	8 BRAM	21,492	10.00	2.15	82,850.00	178.05
					w3a08	1	2834	2612	2384	x	8 BRAM	88,991	10.00	8.90	77,290.00	687.84
						15	2864	6573	4447	x	8 BRAM	12,991	10.00	1.30	95,010.00	123.43
					w3a09	1	3658	3122	2888	x	8 BRAM	61,769	10.00	6.18	80,210.00	495.47
						15	3688	7200	4654	x	8 BRAM	10,545	10.00	1.05	98,620.00	104.00
					w3a10	1	3323	3249	2873	x	7 BRAM	113,098	10.00	11.31	77,480.00	876.23
						15	3337	5238	3738	x	7 BRAM	80,216	10.00	8.02	78,980.00	633.56
					w3a11	1	2005	1768	1551	x	8 BRAM	235,001	10.00	23.50	73,900.00	1736.63
						15	2019	3748	2575	x	8 BRAM	28,230	10.00	2.82	84,820.00	239.45
					w3a12	1	2005	1768	1551	x	8 BRAM	189,372	10.00	18.94	73,860.00	1398.72
						15	2019	3748	2575	x	8 BRAM	23,742	10.00	2.37	86,260.00	204.79
2009	[18]	163	B163	0.13 μm	w4a01	1	x	x	x	16,837	0	169,769	0.50	339.54	16.01	5.44
						2	x	x	x	17,444	0	89,417	0.50	178.83	17.33	3.10
						3	x	x	x	17,957	0	62,633	0.50	125.27	19.98	2.50
						4	x	x	x	18,567	0	48,745	0.50	97.49	22.05	2.15
						8	x	x	x	20,678	0	28,905	0.50	57.81	28.03	1.62
						15	x	x	x	24,561	0	18,985	0.50	37.97	34.63	1.32
						19	x	x	x	26,777	0	17,001	0.50	34.00	41.51	1.41
						55	x	x	x	47,247	0	11,049	0.50	22.10	68.23	1.51
2010	[26]	163	BE163	0.13 μm	w5a01	1	x	x	x	11720	84 bytes	219,148	0.40	547.87	7.27*	3.98
						2	x	x	x	12,348	84 bytes	113,428	0.40	283.57	9.10*	2.58
						3	x	x	x	12,862	84 bytes	78,112	0.40	195.28	10.19*	1.99
						4	x	x	x	13,427	84 bytes	59,800	0.40	149.50	12.00*	1.79
						5	x	x	x	13,970	84 bytes	49,336	0.40	123.34	12.69*	1.57
						6	x	x	x	14,530	84 bytes	42,796	0.40	106.99	13.80*	1.48
2012	[27]	163	B163	0.25 μm	w6a01	1	x	x	x	24140	0	165000	10.00	16.50	5940.00	98.01
						2	x	x	x	24,742	0	84,900	10.00	8.49	7180.00	60.96
						4	x	x	x	26,156	0	44,200	10.00	4.42	8640.00	38.19
						8	x	x	x	31,333	0	23,500	10.00	2.35	13,200.00	31.02
						16	x	x	x	34,956	0	13,500	10.00	1.35	17,400.00	23.49
2016	[32]	163	K163	0.13 μm	w7a01	1	x	x	x	10,106	RAM/ROM	-	1.13	-	36.63	9.16
						2	x	x	x	11,383	RAM/ROM	-	0.59	-	21.55	5.39
						3	x	x	x	12,236	RAM/ROM	-	0.41	-	15.75	3.94
						4	x	x	x	12,863	RAM/ROM	-	0.32	-	12.08	3.02
						5	x	x	x	13,497	RAM/ROM	-	0.27	-	11.41	2.85

Markers: (*) dynamic power; (-) data not available; (x) does not apply. Some results were retrieved from graph representations.

**Table 9:** Adjusted coefficients for the efficiency model of each configuration. The R-square result is provided for the adjustment of the hardware and the energy consumption curves.

Year	Ref.	*m*	Curve	Platform	Conf.	$\alpha_{1}$	$\alpha_{2}$	R-square	$\alpha_{3}$	$\alpha_{4}$	R-square
2006	[22]	131	B131	0.13 μm	w1a01	463.2000	3986.0000	99.99%	9.4985	−0.9107	99.99%
		139	B139			491.7000	4228.5000	99.98%	11.1861	−0.9118	99.99%
		151	B151			528.4000	4593.0000	99.98%	13.7842	−0.9122	99.97%
		163	B163			573.4000	4956.0000	99.99%	16.7408	−0.9169	99.97%
2007	[25]	163	B163	xc3s1000l	w2a01	78.6667	2462.3333	100%	339.6200	−0.0763	100%
					w2a02	69.0667	2622.9333	100%	610.8200	−0.5595	100%
					w2a03	67.0000	1484.0000	100%	549.7400	−0.6610	100%
					w2a04	79.1333	2461.8666	100%	287.0900	−0.0031	100%
					w2a05	79.1333	2461.8666	100%	472.7700	−0.6908	100%
					w2a06	77.6000	1465.4000	100%	333.5100	−0.6503	100%
					w2a07	68.5333	2964.4666	100%	322.5100	−0.1001	100%
					w2a08	75.1333	2548.8666	100%	631.5900	−0.6730	100%
					w2a09	78.6667	1562.3333	100%	471.5500	−0.6538	100%
2009	[17]	163	B163	xc3s500e	w3a01	61.7857	2811.2142	100%	973.1800	−0.1184	100%
					w3a02	73.1429	1477.8571	100%	2070.6300	−0.7317	100%
					w3a03	73.1429	1477.8571	100%	1666.5800	−0.7183	100%
					w3a04	65.0000	2808.0000	100%	858.7000	0.0030	100%
					w3a05	73.1429	1477.8571	100%	1267.9300	−0.7257	100%
					w3a06	61.7857	2811.2142	100%	1288.4500	−0.7229	100%
					w3a07	73.1429	1477.8571	100%	1267.5900	−0.7248	100%
					w3a08	147.3571	2236.6428	100%	687.8400	−0.6344	100%
					w3a09	126.1429	2761.8571	100%	495.4700	−0.5765	100%
					w3a10	61.7857	2811.2141	100%	876.2300	−0.4678	100%
					w3a11	73.1429	1477.8571	100%	1736.6300	−0.7317	100%
					w3a12	73.1429	1477.8571	100%	1398.7200	−0.7095	100%
2009	[18]	163	B163	0.13 μm	w4a01	562.4282	16,236.0223	99.99%	4.9753	−0.5094	89.25%
2010	[26]	163	BE163	0.13 μm	w5a01	556.6000	11,194.0000	99.95%	3.9404	−0.5794	99.50%
2012	[27]	163	B163	0.25 μm	w6a01	752.5578	23,599.5416	95.70%	95.7553	−0.5840	98.33%
2016	[32]	163	K163	0.13 μm	w7a01	826.2000	9538.3999	97.40%	9.1506	−0.7636	99.80%
					C2	110.8941	4050.2750	99.54%	1846.3496	−0.9634	99.92%
2019	This work.	251	BE251	xc6slx16	C3	114.1824	4331.0750	99.17%	1725.1535	−0.9542	99.95%
					C5	115.8118	5409.7250	97.91%	827.4241	−0.9371	99.76%
